# Amygdala size in amyotrophic lateral sclerosis without dementia: an *in vivo *study using MRI volumetry

**DOI:** 10.1186/1471-2377-6-48

**Published:** 2006-12-25

**Authors:** Elmar H Pinkhardt, Ludger Tebartz van Elst, Albert C Ludolph, Jan Kassubek

**Affiliations:** 1 Department of Neurology, University of Ulm, Ulm, Germany; 2 Department for Psychiatry, Albert-Ludwigs-University, Freiburg, Germany

## Abstract

**Background:**

Evidence for extra-motor involvement in non-demented patients with amyotrophic lateral sclerosis (ALS) has been provided by multiple studies, in particular neuropathological studies have demonstrated neuronal loss in the amygdala. The aim of this study was to investigate possible alterations of amygdala volumes *in vivo*.

**Methods:**

Twenty-two moderately disabled patients with definite ALS without cognitive or behavioural deficits and 22 age-matched healthy controls were included. Amygdala and total brain volumes were measured by region-of-interest-based volumetry in 3-D MRI.

**Results:**

A trend was observed with reduced amygdala size in the ALS group, since mean absolute and brain size-corrected amygdala volumes were 6.9% and 7.6% lower in the patient group compared to those in normal controls (P = 0.086 and P = 0.110), respectively.

**Conclusion:**

Volumetrically identifiable alterations of the amygdala can be mapped *in vivo *and may be associated with psychopathological findings in later stages of ALS.

## Background

Amyotrophic lateral sclerosis (ALS) is characterised by a progredient degeneration of upper and lower motor neurons, but there is growing evidence for extra-motor involvement in non-demented patients with ALS that has been provided in neuropathological, neurophysiological, electrophysiological, and neuroimaging studies [1-4]. Not only is the association of motor neuron diseases (MND) with dementia of frontotemporal type well-established, but also the finding of frontotemporal cortex atrophy has been reported at autopsy of patients with the clinical diagnosis of MND without clinical signs of dementia [4]. However, neuropathological extra-motor alterations are not limited to neocortical regions. Ubiquitin immunoreactive inclusions, as they are observed in lower motoneurons in ALS [5], were also found in neurons of the amygdala, in particular the baso-lateral aspect, in more than 30% of cases out of a group of non-demented MND patients [6]. Moreover, animal experiments in transgenic mice of a functionally altered glutamate AMPA receptor subunit (GluR-B(N)) which can result in a phenotype characterized by a pattern of neurodegeneration and progressive motor decline resembling MND in humans, exhibited a selective loss of neurons in the amygdala [7]. The amygdala is known to be an important element of the limbic system, so its involvement may be an important factor for behavioural changes or particularly for altered emotional responses in ALS without dementia, as they have been demonstrated in a previous study [8].

*In vivo *investigations of pathological changes using high-resolution magnetic resonance imaging (MRI) raise the possibility of analysing the topography of volume alterations. So far, there are no studies specifically investigating amygdala pathology in MND using modern MRI techniques. In this MRI-based volumetric study, we tested the hypothesis that there is amygdala volume loss in patients with ALS by a comparison with healthy subjects.

## Methods

### Patients and controls

The study was approved by the local ethics committee (Ethics Committee of the University of Ulm), and all patients gave their written informed consent. Twenty-two patients with a clinical diagnosis of ALS according to the revised El Escorial criteria for the diagnosis of ALS [9] were included in the study (mean age 58 ± 9 years; 19 men, 3 women). The MRI data of the same patients and the controls had been previously analysed by automated techniques (voxel-based morphometry) in which no volume alterations of the amygdala were observed in a whole brain study at group level [10]. All patients had clinical evidence of upper motor neuron degeneration as well as clinical and electrophysiological evidence for lower motor neuron involvement in at least three regions, as shown by independent investigations by two experienced neurologists at the time of scanning. Nineteen patients had limb onset, three had bulbar onset. The mean time between onset of motor symptoms (as assessed by patients' self-reports) and MRI scanning was 2 years. The ALS Functional Rating Scale [11] ranged from 20 to 40 (median 30). A neuropsychological test battery was performed including tests for frontal functions (Regensburg Fluency Test for verbal fluency or 5-Point Fluency Test for design fluency), Symbol Digit Modalities Test, Wisconsin Card Sorting Test (PC version), Doors Test, and Beck's Depression Inventory (BDI). Here, design fluency, planning and conceptual formation, attention, and visual memory were unimpaired in all patients, verbal fluency was normal in all but two patients. The patients scored a mean of 7 (range 4–16) in BDI, only two patients fulfilled the criteria of mild depression; thus, none of the patients met the cutoff for moderate depression of 18. There were no behavioural abnormalities in all patients.

A control cohort was set up by acquiring MRI data from an age-matched group of 22 healthy subjects (mean age 59 ± 11 years; 17 men, 5 women) by use of the same scanning protocol. All volunteers were neurologically and neuropsychologically normal (as shown by the use of the same test battery), none had any history of neurological or psychiatric disease or other medical conditions.

### Data acquisition and processing

High-resolution MRI was acquired in all subjects on the same 1.5 Tesla clinical scanner with a standard quadrature headcoil (Siemens, Erlangen, Germany). Three-dimensional data sets of the whole brain were collected using a T1 weighted magnetisation prepared rapid acquisition gradient echo (MP-RAGE) sequence, consisting of 160 to 180 sagittal partitions depending on head size. The acquisition parameters were as follows, repetition time 9.7 ms; echo time, 3.93 ms; flip angle, 15°; matrix, 256 × 256 mm^2^; field of view, 250 mm.

**Figure 1:**
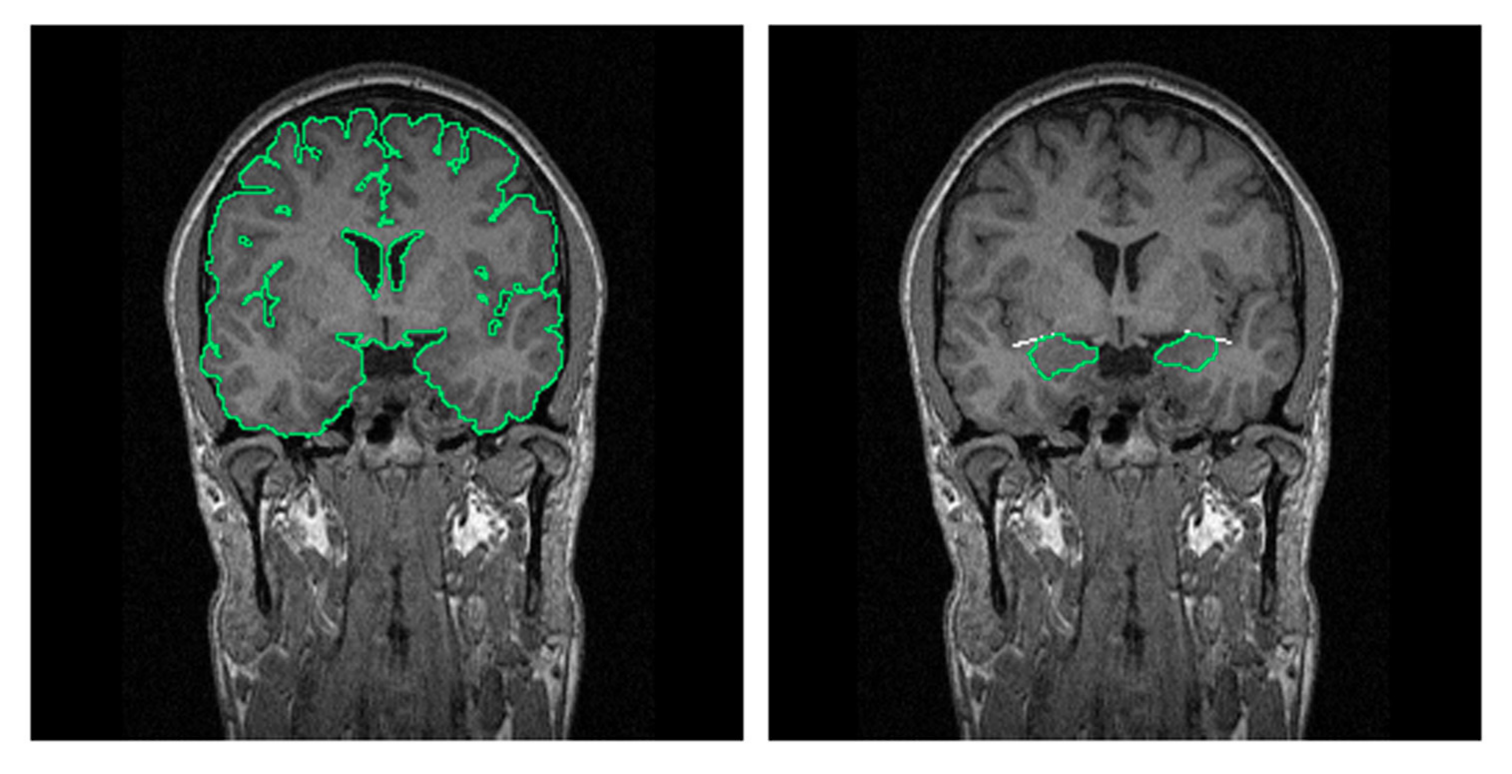
Examples of single 3-D MRI slices, showing delineation of in-slice volumes of total brain (left) and amygdala (right) [green] and the defined superior limit according to Watson et al. [14] [white] within *MRreg*.

All MRI data were processed by use of the interactive software program *MRreg *(L. Lemieux, Epilepsy Imaging Group, Dept. of Clinical and Experimental Epilepsy, Institute of Neurology, UCL, London, UK [12]) which enables volumetric measurements by manual delineation of specific intracerebral structures. For assessment of the total brain volume, the brain structures were manually outlined in every tenth slice at a 2 times magnification using the semi-automatic threshold and region growing tool to delineate cerebrum, cerebellum, upper brainstem according to [13]. The resulting voxel number was multiplied by the voxel volume to give the in-slice brain volume (cm^3^), and the total brain volume was then obtained by multiplication of the sum of the in-slice volumes by 10. For outlining the amygdala, the images were zoomed to a 4 times magnification. The volumes were then measured by delineating the amygdala boundaries for each hemisphere separately, following the established protocol described by Watson et al. [14]. The in-slice volume (i.e., the volume of the delineated amygdala in each slice) was calculated by multiplying the number of voxels contained within each trace by the voxel volume and dividing this value by the magnification factor 4. The sum of all in-slice volumes resulted in the total volume of each amygdala. Representative slices showing total brain and amygdala delineation are given in Figure 1. For calculation of the relative amygdala size, the amygdala volumes were divided by the total brain volume of the subject, providing the proportion amygdala/total brain and thus a correction for individual brain size [15].

The rater was trained in volume-of-interest-based analysis of MRI and was blinded to the subject grouping. Intrarater reliability was tested by repeated measurements of a subset of 10 subjects.

Patient and control groups were compared using the t-test within the Statistical Package for the Social Sciences software (SPSS, Version 12.0, Chicago, IL, USA). A two-sided level of significance of 0.05 was defined. The intrarater reliability was assessed by four different methods: (i) the ratio of measured standard deviations to average amygdala volumes (coefficient of variation), (ii) the coefficient of repeatability, C_r _[16], (iii) an intraclass correlation coefficient, and (iv) the similarity index (SI) in order to compute measures of overlap of the ROIs according to Heckemann et al. [17,18].

## Results

### Volumetric measurements

**Figure 2:**
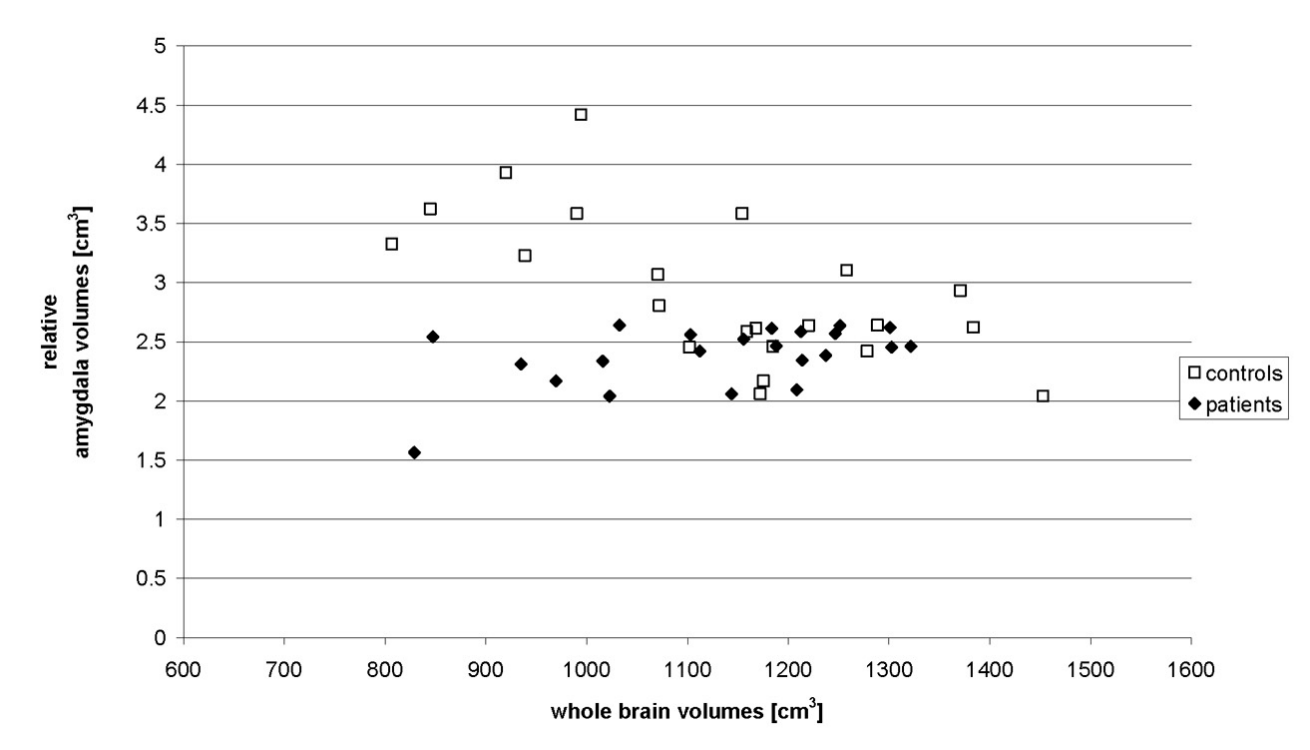
Relative amygdala volumes plotted against total brain volumes in ALS patients and controls.

Table 1 summarises the volumetric findings. Both for left and for right amygdala, the patients' mean volumes were lower than the controls', with no significant asymmetry effect (left vs. right amygdala volumes, p = 0.212 in patients and p = 0.335 in controls). The comparison of uncorrected right-sided amygdala volumes between patients and controls was statistically significant, but unilateral findings or amygdalar asymmetry was not included in our hypothesis-driven approach. The total brain volumes were 1,127.7 ± 140.1 cm^3 ^in patients and 1,135.6 ± 167.7 cm^3 ^in controls (p = 0.440). The total amygdala volumes, both as the absolute value and corrected for brain size, were lower in the ALS patients. However, this finding has to be judged as a trend since it failed to reach statistical significance (p = 0.086 for absolute and p = 0.110 for relative volumes, respectively). Corrected amygdala volumes plotted against total brain volumes are shown in Figure 2.

### Reliability data

For intrarater reliability, the coefficient of variation was 8.5%. The coefficient of repeatability (Cr) was 224 mm^3^, i.e. 14% of the overall mean, and the intraclass correlation coefficient was 0.65. All these values corresponded well to what has been reported in the literature [19]. The mean SI was 0.793 ± 0.038 (range 0.736–0.849) for the right amygdala and 0.783 ± 0.029 (range 0.723–0.843) for the left amygdala. These values also corresponded well to previous studies [17].

## Discussion

The volumetric 3-D MRI-based measurements of amygdala demonstrated a trend for volume reduction in patients with clinically definite ALS, i.e. a mean decrease by 6.9% in absolute and 7.6% in corrected values. These data are in general accordance with autopsy studies which describe intraneuronal inclusion bodies and marked gliosis in the amygdala of ALS patients [6], although it has to be held that these histological changes might not influence volumes per se. Another reason for the apparent discrepancy between the marked amygdala alterations in neuropathological studies and the non-significant findings in MRI-based studies might be that the latter including the present one were performed in patients who were moderately disabled and that amygdala changes might be more pronounced in the later stages. The neuropathological findings seem to be more common in ALS patients with dementia [2], but they are not limited to these cases and are found in non-demented ALS patients as well [6]. The lower volumes in ALS are obviously a subtle finding in *in vivo *studies which was only detected when amygdala volumes were specifically addressed – in contrast, there were no amygdala changes found in previous whole brain-based investigations in ALS using statistical comparisons of 3-D MRI data (voxel-based morphometry), neither in non-demented ALS patients [10] nor in patients suffering from ALS/frontotemporal lobar degeneration complex [20]. The trend for lower amygdala volumes is the more remarkable since there was no global atrophy of the ALS patients' brains found here (i.e., the total brain volumes did not differ from controls), opposite to previously used automated techniques [10]. These discrepant findings might be explained by the basic methodological differences in global atrophy assessment between the two studies. In the present study, a volume-of-based approach was used on 3-D MRI data in native space to determine total brain volumes, for the purpose of correcting absolute amygdala volumes for global brain atrophy in a ratio of absolute volumes. In the previous study [10], brain parenchymal fractions (BPF) were investigated, i.e. size-normalised proportions of grey matter and white matter volumes to total intracranial volumes, calculated after pre-processing steps including statistical parametric mapping (SPM)-based spatial normalisation to standard space and automatic segmentation. Obviously the manual ROI delineation and automatic growing tool, performed in every tenth slice, within *MRreg *on the one hand and the SPM-based automatic processing of normalised data on the other hand produced different results with respect to the statistical significance of the comparison of ALS patients and controls.

The functional impact of the subtle structural amygdala abnormalities is hard to assess. The amygdala is believed to be important among other functions for evaluation of the emotional valence of stimuli in the early phases of sensory processing (assigning emotional significance to specific sensory input [21]), linkage of perceptual representation to emotional memories and regulation of autonomic responses [22]. In humans, the amygdala appears to be especially important for processing of emotional information in a social context [23]. Affection and emotional processing in ALS is poorly understood beyond the general agreement that ALS patients do not develop a depressive disorder despite the severe impact of the disease on their lives. It has been demonstrated that emotional responses of ALS patients tend to be altered towards positive valence [8], and it seems a plausible concept that alterations of the amygdala might be involved in this process.

Whether the volume reduction implies *per se *a loss of function of the amygdala or is just the morphological correlate of a generally altered function, remains unresolved. According to a PET study, a group of non-demented MND patients had a reduced regional cerebral blood flow in limbo-thalamic areas [24], indicative of functional decrease. With respect to cognitive abilities, the division of human behaviour into emotion and cognition is not as clear as previously suggested, and the finding that brain regions linked to executive function and working memory are correlated with amygdala response suggests one pathway by which complex cognitive manipulations of stimuli might influence the neural circuitry of emotion, so for instance an indirect communication between frontal cortical areas and amygdala exists [25]. The involvement of amygdala volume decreases in dementia, even as a predictive factor, has been demonstrated, however, only for Alzheimer disease yet [26], but might be part of the ALS/dementia complex as well.

## Conclusion

In summary, a trend for volume alterations of the amygdala in ALS could be observed *in vivo *by use of MRI volumetry. Additional investigations in later stage patients and patients with dementia and/or distinctive behavioural deficits promise further insights in the role of limbic system involvement within the multi-system degeneration process of MND.

## Competing interests

The author(s) declare that they have no competing interests.

## Authors' contributions

EHP and JK drafted the manuscript, LTvE and ACL revised it critically for important intellectual content. JK and ACL designed the MRI study, JK was involved in coordination of the study. EHP and JK performed the data acquisition and pre-processing of the data. LTvE participated in the pre-processing and the statistical analysis of the data. EHP performed the measurements and the statistical analysis. JK and ACL were involved in the interpretation of results and general conclusions. All authors read and approved the final manuscript.

## Pre-publication history

The pre-publication history for this paper can be accessed here:



## Figures and Tables

**Table 1:** Synopsis of volumetric findings in ALS patients and controls (mean values ± SD).

	right amygdala volume [cm^3^]	left amygdala volume [cm^3^]	total amygdala volume [cm^3^]	proportion of amydala to total brain volume [× 10^-3^]
patients	1.487 ± 0.262	1.526 ± 0.312	3.014 ± 0.534	2.69 ± 0.609
controls	1.628 ± 0.258	1.610 ± 0.288	3.239 ± 0.512	2.91 ± 0.488
t-test	P = 0.043	P = 0.186	P = 0.086	P = 0.110
